# DNA Methylation of the First Exon Is Tightly Linked to Transcriptional Silencing

**DOI:** 10.1371/journal.pone.0014524

**Published:** 2011-01-18

**Authors:** Fabienne Brenet, Michelle Moh, Patricia Funk, Erika Feierstein, Agnes J. Viale, Nicholas D. Socci, Joseph M. Scandura

**Affiliations:** 1 Laboratory of Molecular Hematopoiesis, Department of Medicine, Weill Cornell Medical College, New York, New York, United States of America; 2 Leukemia Program, Weill Cornell Medical College, New York, New York, United States of America; 3 Genomics Core Laboratory, Memorial Sloan-Kettering Cancer Center, New York, New York, United States of America; 4 BioInformatics Core, Memorial Sloan-Kettering Cancer Center, New York, New York, United States of America; The Rockefeller University, United States of America

## Abstract

Tissue specific patterns of methylated cytosine residues vary with age, can be altered by environmental factors, and are often abnormal in human disease yet the cellular consequences of DNA methylation are incompletely understood. Although the bodies of highly expressed genes are often extensively methylated in plants, the relationship between intragenic methylation and expression is less clear in mammalian cells. We performed genome-wide analyses of DNA methylation and gene expression to determine how the pattern of intragenic methylation correlates with transcription and to assess the relationship between methylation of exonic and intronic portions of the gene body. We found that dense exonic methylation is far more common than previously recognized or expected statistically, yet first exons are relatively spared compared to more downstream exons and introns. Dense methylation surrounding the transcription start site (TSS) is uncoupled from methylation within more downstream regions suggesting that there are at least two classes of intragenic methylation. Whereas methylation surrounding the TSS is tightly linked to transcriptional silencing, methylation of more downstream regions is unassociated with the magnitude of gene expression. Notably, we found that DNA methylation downstream of the TSS, in the region of the first exon, is much more tightly linked to transcriptional silencing than is methylation in the upstream promoter region. These data provide direct evidence that DNA methylation is interpreted dissimilarly in different regions of the gene body and suggest that first exon methylation blocks transcript initiation, or vice versa. Our data also show that once initiated, downstream methylation is not a significant impediment to polymerase extension. Thus, the consequences of most intragenic DNA methylation must extend beyond the modulation of transcription magnitude.

Sequencing data and gene expression microarray data have been submitted to the GEO online database (accession number SRA012081.1). Supporting information including expanded methods and ten additional figures in support of the manuscript is provided.

## Introduction

The human genome is adorned with methylated cytosine residues that function in the epigenetic guidance of cellular differentiation and development. Regional DNA methylation patterns are initially established during early embryogenesis and subsequently remodelled in differentiating cells [1], [2], [3], [4]. DNA methylation is essential for normal development, genomic imprinting and X chromosome inactivation, and functions in the silencing of transposable elements and, perhaps, in the maintenance of genomic integrity [5], [6], [7]. Despite the breadth of these activities, our understanding of the epigenetic machinery governing DNA methylation and its effects is incomplete.

Vertebrate DNA methyltransferases (DNMTs) act upon cytosines in the context of the cytosine-phospho-guanosine dinucleotide (CpG). Particular histone modifications, such as those placed by polycomb repressive complexes (PRCs), are associated with the site-specific recruitment of DNMTs [8], [9], [10]. In turn, methyl-CpG serves as the physiologic ligand for a family of proteins containing a highly conserved, methyl-CpG binding domain (MBD) [11]. The MBD sequence motif folds as a structural domain that exclusively binds methylated CpGs via narrow interactions between the methyl-CpG dinucleotide and a hydrophobic patch within the MBD domain [12], [13]. MBD-containing proteins (MBPs) recruit various chromatin-modifying complexes to methyl-CpG sites to bring about further changes in chromatin structure: prototypically those associated with nucleosomal compaction and transcriptional silencing.

The linkage between gene promoter methylation and heritable transcriptional suppression is well recognized, but the function of intragenic DNA methylation is more obscure [1], [14], [15], [16], [17]. Methyl-CpGs dominate mammalian genomes and extensive methylation within the body of coding genes is common in both plants and animals [4], [18], [19], [20]. The vast majority of this methylation occurs in regions of low CpG density (∼1 CpG per 100 bp) [4], [21] yet interspersed in this sea of low-density methylation are select regions such as CpG islands (CGIs) with higher CpG content and more variable methylation [1]. In contrast to promoter methylation, the relationship between gene body methylation and transcription is less well established and may differ in mammals and plants, at least when this intragenic methylation is considered as a *composite* of all methylation occurring between the start of the first exon and the end of the last exon [4], [18], [19], [20], [22]. These prior composite analyses do not accommodate differential functions for *regional* intragenic methylation yet the distinct roles of introns and exons suggest that the biological significance of methylation within these elements may differ. Furthermore, the outcome of genic methylation may be linked to the density of CpG methylation as this has proven to be closely associated with transcriptional silencing in the context of promoter methylation [23], [24], [25], [26].

To advance these prior composite studies, we investigated the cross-correlation between DNA methylation within different regions of the gene cassette (promoter, first exons, introns, internal exons and last exons) and we assessed how these different classes of regional methylation are associated with transcription. We utilized a technology that is sensitive to the density of CpG methylation and found that densely methylated elements (DMEs) of the genome are disproportionately enriched for exons. We found that methylation within introns and downstream exons is highly correlated but uncoupled from methylation surrounding the transcription start site (TSS) and most divergent from methylation within the first exon. Methylation at the 5′ end of a gene was associated with transcriptional silencing whereas methylation in the more downstream portions of the gene body was not. Most strikingly, we found that even modest transcription was strictly associated with low first exon methylation. In contrast, the linkage between gene expression and upstream promoter methylation was more variable and less stringent. These data point to divergent functions for methylation within different regions of the gene body and suggest that methylation of the first exon is critical for transcriptional silencing.

## Results

### Genome-wide identification of densely methylated elements

**Figure 1:**
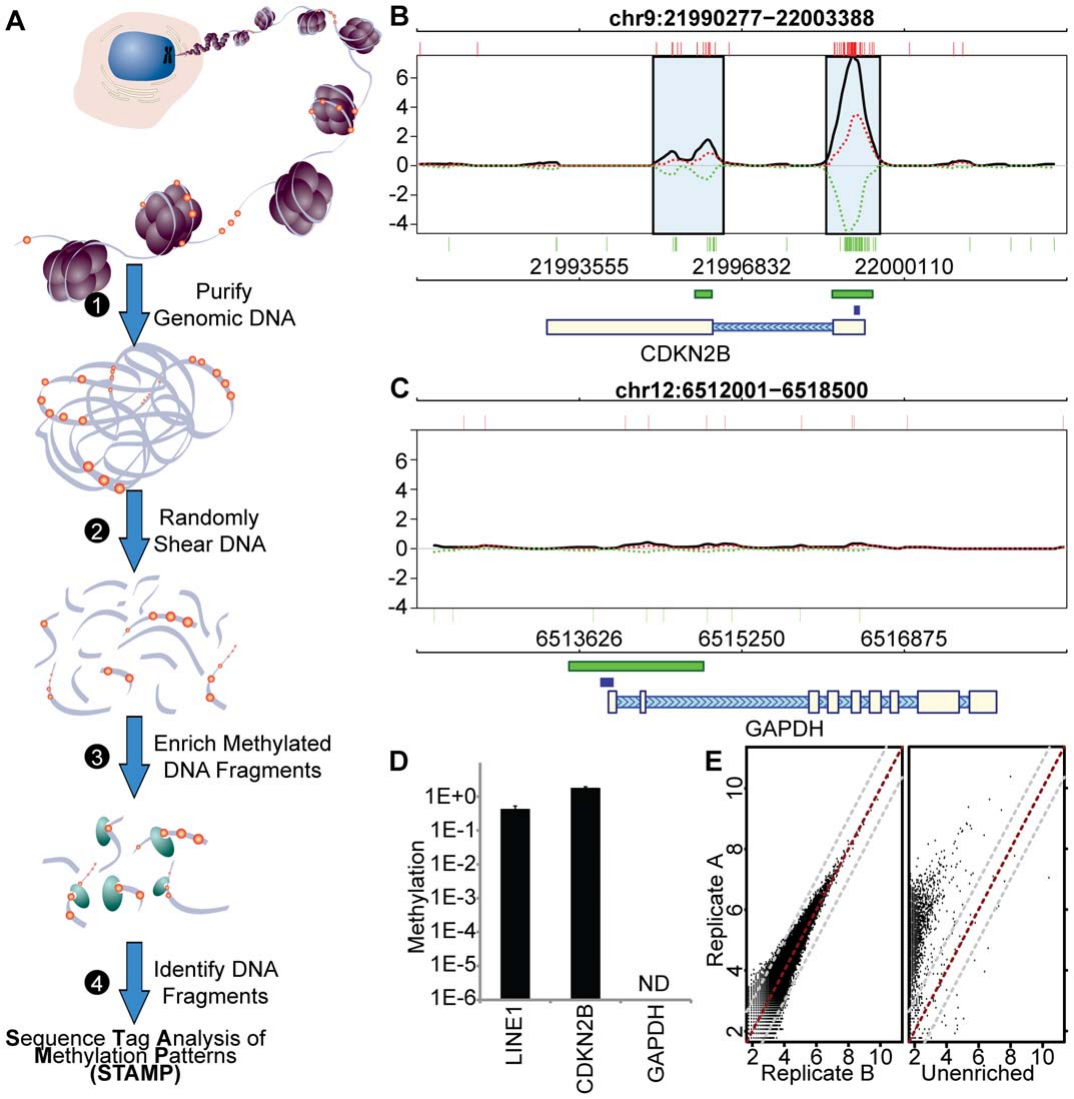
Overview of the STAMP assay and representative data. (**A**)The steps involved in the preparation of a methylated DNA library for massively parallel sequencing is illustrated schematically. 1) Genomic DNA is purified from cells. 2) The DNA is then randomly sheared by sonication. 3) Fragmented DNA containing methyl-CpGs, indicated as red spheres, is purified using His-MBD beads. 4) Bound DNA is purified and ligated to SOLiD sequencing adapters. The resulting DNA library is subsequently sequenced and mapped to the genome of interest. These sequence tags inform subsequent analysis of DNA methylation patterns. (**B**) Sequence tag maps and methylation profile is shown at the CDKN2B locus for the AML cell line M091. Upper, red vertical bars and lower, green vertical bars represent individual sequence tags mapping to the sense and antisense strands, respectively. The dashed red and green lines represent the methylation signal for the top strand and bottom strand, respectively. The black line represents the composite STAMP signal. A light blue box surrounds each densely methylated element (DME). CpG Islands are shown as green boxes below the plot and the gene body is indicated schematically. The location of the PCR amplicon used for bisulfite qPCR is indicated by a blue box. (**C**) STAMP analysis at the GAPDH locus, as described for panel (B), shows no methylation at this locus. STAMP analysis at the CDKN2B and GAPDH loci was confirmed by (**D**) Bisulfite qPCR (Methylight) (see Supporting Information S1, Table 1). In this panel, the fraction of total DNA present (assessed using methylation-insensitive primers) that is detected as methylated is shown. (**E**) STAMP analyses of biological replicate cultures of the AML cell line is shown in the left panel scattergram. A STAMP signal (log scale) for the replicates was calculated at 15,000 randomly selected loci. The red and grey dashed lines represent unchanged and two-fold changed signal The right panel compares one of the replicates to sequence tags obtained from unenriched DNA from the same cell line and demonstrates that the high replicate correlation depends upon His-MBD enrichment.

To study genome-wide methylation patterns, we developed a method that leverages the selectivity of the MBD with the breadth and flexibility of massively parallel sequencing using the SOLiD sequencer (**Fig. 1A**). We optimized this Sequence Tag Analysis of Methylation Patterns (STAMP) assay for robust, whole-genome identification of methylated DNA segments. We expressed a His-tagged fragment of MBD1 (aa 1–69) in bacteria to generate an affinity matrix. This fragment (His-MBD) contains the critical MBD domain contacts required for stable and selective binding to methyl-CpG but no structural elements known to contribute to sequence-specific DNA binding (**Supporting Information S1**) [13]. The His-MBD fragment was collected on IMAC superparamagnetic polystyrene beads (Dynabeads Talon, Invitrogen) and used for microscale purification of randomly sheared (<200 bp) methylated DNA in the STAMP assay. We performed STAMP analysis of a human leukemia-derived cell line, M091 [27] and evaluated STAMP results at loci that we knew to be transcriptionally silenced, (CDKN2B, p15INK4b), or robustly expressed (GAPDH). We found highly clustered sequence tags (tags) mapping to the sense strand (red vertical bars) and antisense strand (green vertical bars) at the silenced CDKN2B locus (**Fig. 1B**) [27]. In contrast, far fewer tags with no apparent clustering were found at the GAPDH gene locus (**Fig. 1C**). We used the tag maps to infer a methylation signal (black solid line) from the superposition of the top-strand signal (red dashed line) and the bottom strand signal (green dashed line) (**Supporting Information S1**). From these data, we identified Densely Methylated Elements (DME) with algorithms we developed to ensure a low false discovery rate across the genome (see **Methods**). As validation, we performed qPCR on bisulfite-treated DNA (Methylight) using the LINE1 promoter consensus sequence as a positive control (**Fig. 1D**) [28], [29], [30], [31]. We also performed deep bisulfite sequencing of amplicons spanning the CDKN2B locus (**Supporting Information S1**). These results confirmed the methylation state of CDKN2B and GAPDH and demonstrated the specificity of the STAMP assay.

### STAMP assay is highly reproducible and sensitive to methylated CpG density

We performed several analyses to assess the performance of the STAMP assay. First, we compared the STAMP methylation signal in biological replicates and found that the signal was highly correlated despite there being very few (∼2.7%) sequence tags common to both data sets (**Fig. 1E**). No such correlation was identified between sequence tags of His-MBD enriched and unenriched DNA isolated from the same cells (**Fig. 1E, right panel**). To assess the ability of the STAMP assay to discriminate similar specimens, we performed replicate analyses using DNA isolated from M091 cells that were either untreated or treated with the hypomethylating agent, 5-aza-2′-deoxycytidine (decitabine). This is a stringent test because the two samples share virtually all methylated loci, differing predominantly in the scale of the methylation signal. We calculated a STAMP signal at 15,000 random genomic locations and for each pair of samples we plotted the log ratio of the samples (M) versus the average log signal (A) at each locus (**Supporting Information S1**). These MA plots demonstrate the high reproducibility of biological replicates and reveal a systematic difference in scale when the STAMP signal from untreated cells is compared to that of cells treated with decitabine.

Next, we compared the STAMP signal at 27,578 CpGs interrogated by direct bisulfite analysis using the Illumina HumanMethylation27 microarray (**Supporting Information S1**). We found a log-linear relationship between the STAMP signal and fractional methylation reported by the Illumina array. To assess the relationship between STAMP signal and CpG density, we separated probes into 11 bins based upon the CpG density surrounding the CpG interrogated by each probe. The log-linear correlation between the STAMP signal and fractional methylation was maintained at all but the lowest CpG densities (<0.02) with the relationship being relatively constant when the CpG density was ≥0.05 (slope = 3.9, r = 0.82). The STAMP signal had a broad dynamic range owing to its reporting regional methylation rather than fractional methylation at a single CpG. Thus, the STAMP assay provides highly reproducible, specific data that are dependent upon DNA methylation density and that can be used to differentiate very similar specimens.

### Distinct classes of intragenic methylation

**Figure 2:**
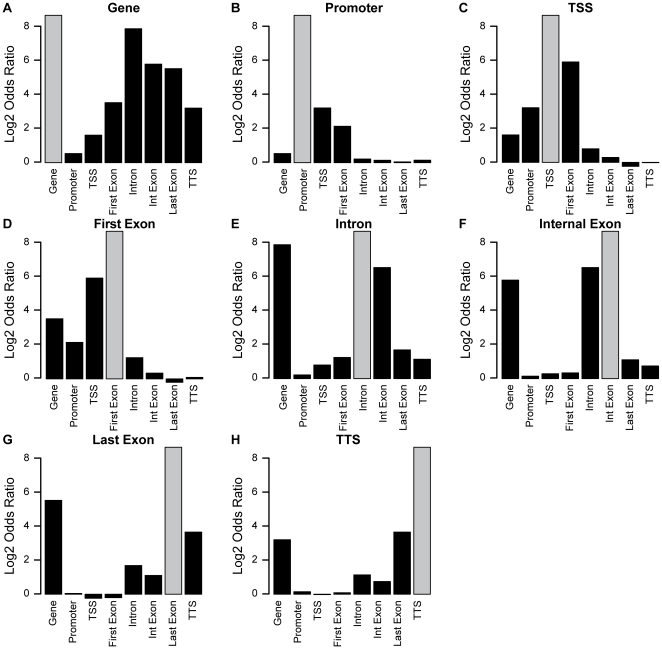
Patterns of gene cassette methylation in T cells. Each gene cassette element was classified as unmethylated (lowest 10% methylation quantile) or methylated (top 90% methylation quantile). The odds ratio (log2 transformed) indicates the likelihood of an element being methylated if the gene body (A), promoter (B), TSS (C), first exon (D), any intron (E), any internal exon (F), last exon (G) or TTS (H) is methylated. Odds ratios, calculated using Fisher's exact test for count data, represent a conditional maximum likelihood estimate quantifying the strength of the correlation between methylation of each gene cassette element. The odds ratio for the autocorrelation of each element is infinite and represented by grey boxes. Representative data is shown for human blood T cells but the pattern is the same in granulocytes and AML cells.

Recent genome-wide analyses of DNA methylation have not explored how methylation within different elements of a coding unit relate to one another [4], [18], [32], [33]. To investigate these relationships, we quantified DNA methylation within each intron and exon of all transcripts annotated in the Refseq database and in annotations we created for all Refseq promoters (from −1000 bp to TSS), TSS (+/− 250 bp surrounding the TSS) and TTS (+/− 250 bp surrounding the end of the last exon). We counted sequence tags within each of these regions and then divided these counts by the genomic span of each element to generate a pseudo-density that is independent of region length. We ranked these pseudo-densities and classified each element as unmethylated (lowest 10% quantile) or methylated (top 90% quantiles) to generate contingency tables for each component of every gene cassette. We analyzed these tables using Fisher's exact test for count data and calculated a conditional maximum likelihood estimate (odds ratio) to quantify the strength of the correlations between methylation of each component of every transcriptional unit (**Fig. 2**).

This analysis revealed that DNA methylation surrounding the TSS generally diverges from methylation within more downstream intragenic elements. The tight correlation we observed between methylation of the promoter, TSS and first exon can be partly explained by limited overlap of these elements. Similarly, genomic proximity can explain the association between methylation of the TTS and the last exon. However, the 5′ and 3′ gene ends have surprisingly distinct relationships to methylation within the rest of the gene. Gene body methylation as a whole was much more loosely coupled to methylation at the 5′ end than it was with any other constituent part (**Fig. 2A**). Methylation in introns or internal exons (i.e., those that are neither first or last exons) was closely linked to methylation within the 3′ genic elements but not with methylation surrounding the TSS (**Figs. 2E, 2F**). These results suggest that 5′ methylation and methylation within more downstream regions either have distinct functions or are under independent regulatory control.

### Genomic distribution of densely-methylated elements is highly non-random

To assess the genomic distribution of densely methylated elements (DME), we measured DME overlap with each of four UCSC genome annotation tracks: *cpgIslandExt* (CGI: CpG Islands); *phastConsElements17way* (Conserved: 17-way most conserved elements); *refGene* (Promoter, Gene, TSS, Exon-5′, Exon-3′, Exon-In, Intron: for refSeq genes); and *rmskRM327* (Repeat Classes: Repeat Masker). As a control, we also constructed an artificial annotation (Random) comprised of 31,000 randomly selected, 10 Kb genomic windows encompassing ∼10% of the genome. We evaluated the methylation patterns of normal peripheral blood T cells and granulocytes and also used an AML-derived cell line (M091) to evaluate how the DNA hypomethylating agent, 5-aza-2′-deoxycytidine (decitabine), affects these patterns.

**Figure 3:**
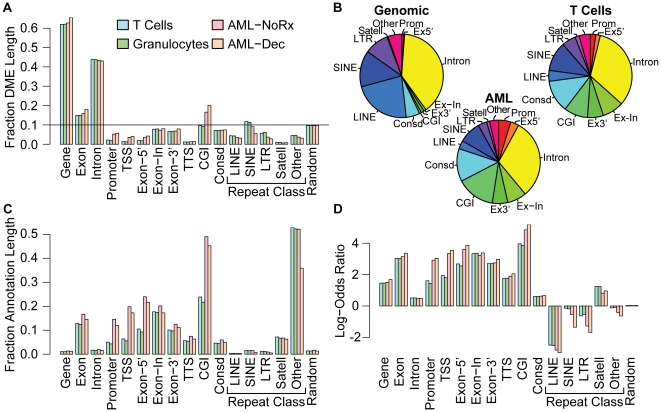
Genomic distribution of Densely-Methylated Elements (DMEs). (**A**) The fraction of the genomic DME span overlapping the indicated UCSC genome browser annotation tracks is shown for normal human T cells (blue bars), granulocytes (green bars) and an AML-derived cell line (M091) that was treated with decitabine (orange bars) or left untreated (pink bars). Gene, Promoter, TSS, TTS, Exon, Intron, Exon-5′, Exon-3′, and Exon-In represent the entire gene body, the region −1000 bp upstream of the TSS, the 500 bp surrounding the TSS or TTS, all exons, all introns, and the first, last or middle exons, respectively. Also annotated are CGIs, the most conserved genomic elements (Consd), various repeat classes and a group of random genomic loci comprising 10% of the genome (Random). The sum of the DME fractions is greater than one because DMEs may hit more than one annotation due to overlap of some genomic annotations. (**B**) The proportion of the genome allocated to each annotation (left panel) is compared to the proportional size of DMEs within each annotation for T cells (right panel) and for the AML-derived cell line (lower panel). For clarity, gene bodies are excluded. (**C**) The fraction of the annotation span overlapping DME is shown for normal human T cells (blue bars), granulocytes (green bars) and an AML-derived cell line that was treated with decitabine (orange bars) or left untreated (pink bars), as described for (A). (**D**) The log-odds ratio for the extent of DME overlap compared to that expected from the relative genomic span of the annotation is shown as described for (A).

In all cell types we evaluated, the distribution of DMEs was highly biased for particular annotations and this bias could not be explained by the size of the genomic annotation (**Fig. 3**). Dense methylation of exonic regions was much more common than expected from the relatively small contribution of exons (∼2%) to genome span (**Fig. 3B** **, Genomic**). CGIs and highly conserved elements were also disproportionately methylated (**Fig. 3B**). The extent to which individual annotations were covered by DMEs (fraction annotation length) varied considerably (**Fig. 3C**) but the overlap of CGIs and exonic regions was most widespread with the exception of first exons, which were relatively spared in normal cell types (**Fig. 3C**). In contrast, we found much more extensive methylation of CGIs and 5′ genic elements in an AML cell line that has a methylator phenotype [34]. The strong bias for CGIs, exons and the most highly conserved regions of the genome could not be explained by chance. We calculated the log-likelihood of a DME hitting an annotation track by comparing the observed DME distribution to that expected from the relative sizes of the annotations (**Fig. 3B** **, Genomic**). Again we found that CGIs, exons and conserved elements were hit far more frequently than expected stochastically (**Fig. 3D**). Microsatellites and RNA repeats (particularly rRNA, included in the “other” repeat class) had the most disproportionate tag enrichment of the repetitive elements. In contrast, several annotations that occupy relatively large portions (introns, LINEs, SINEs) of the genome were underrepresented likely owing to the non-uniformity of methylation in these regions (e.g., LINEs are predominantly methylated within vestigial promoters). Although many repetitive element regions are distinctive enough to permit unique mapping of 35 or 50 bp sequence reads, underrepresentation of repetitive elements in the “mappable” genome may also contribute to the lower than expected overlap between DMEs and several repeat classes. We found that decitabine treatment did not lead to changes in the genomic distribution of DMEs, although the overlap of each annotation by DMEs was reduced (**Fig. 3C**). Taken together these results demonstrate that most DMEs are intragenic and are preferentially concentrated within exons, CGIs and conserved regions of the genome.

### DMEs are not classic CpG islands

**Figure 4:**
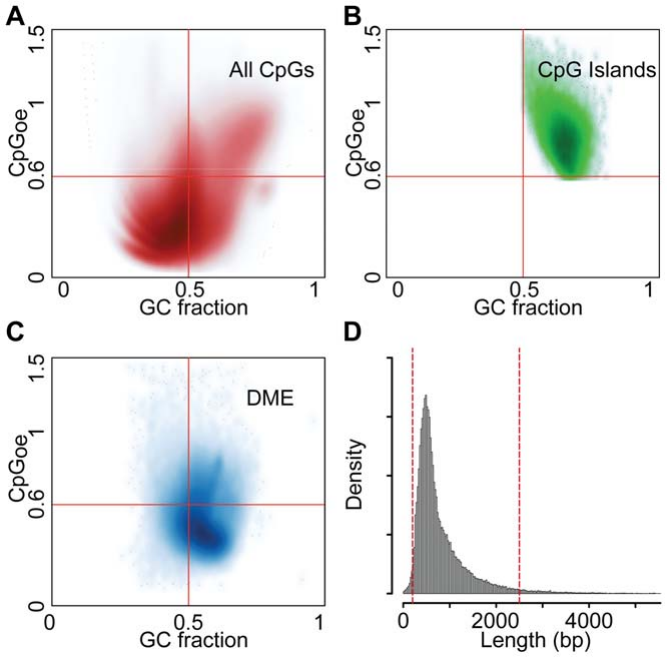
Sequence characteristics of Densely-Methylated Elements (DMEs). (**A**) Density plot of GC fraction (fG+fC) vs CpGoe, the observed/expected CG fraction, fCG/(fC*fG) for a 200 bp window surrounding each CpG dinucleotide in the genome. (**B**) The GC fraction vs. CpGoe is plotted for each annotated CGI in the genome. CGIs are partially defined by GC>0.5 and CpGoe>0.6. (**C**) The sequence characteristics of DMEs are plotted. DMEs are enriched for regions with moderate CpGoe. (**D**) The distribution of DME lengths is shown along with dashed red lines representing the 5^th^ (260 bp) and 95^th^ (2140 bp) percentiles. The median length is 590 bp.

Individual CpG dinucleotides reside within a local sequence context with distinct CpG density and GC fraction (**Fig. 4A**). CGIs are defined as clusters of CpG dinucleotides above particular thresholds of length, CpG frequency (corrected for GC content) and GC content (**Fig. 4B**) [16]. We found that the vast majority of the DMEs are not classical CGIs (**Fig. 4C**). Rather, DMEs are GC-rich regions (median 57% GC) with a greater than expected incidence CpG dinucleotides (median CpG observed/expected: 0.49) and a median length of ∼600 bp (**Fig. 4D**). The longest DMEs, which are predominantly microsatellite clusters, extend up to 24,000 bp but 75% of them are less than 960 bp. These results suggest that the definition of CGI excludes the majority of the densely methylated human genome.

### Patterned DNA methylation at the 5′ and 3′ ends of genes

**Figure 5:**
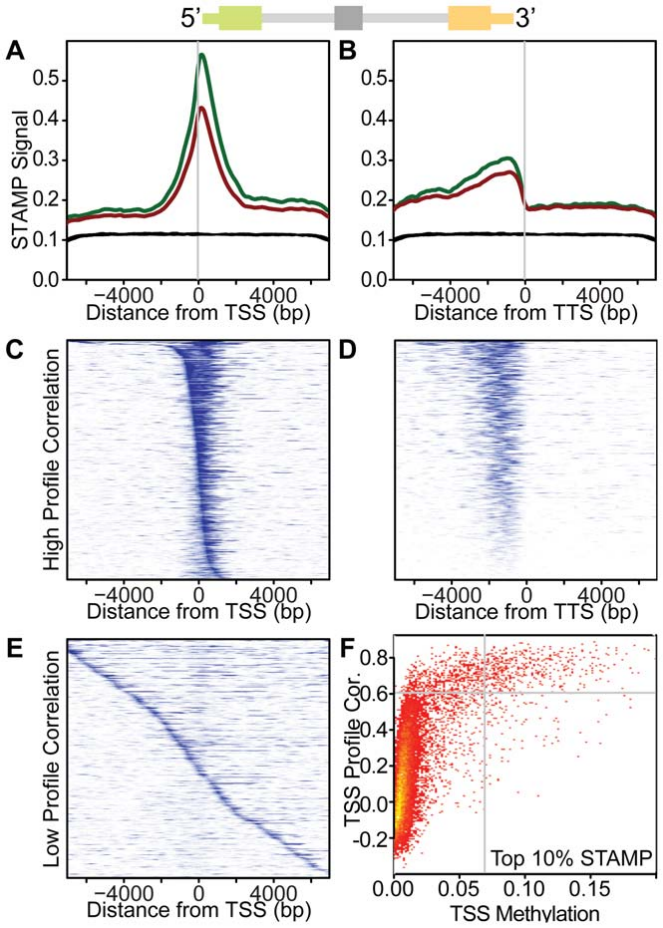
STAMP signal at the TSS and TTS for refSeq genes (refGene). Composite density plots reveal the pattern of STAMP methylation surrounding all TSS (**A**) or TTS (**B**) in M091 cells. STAMP signal was calculated for each TSS or TTS flanked by ∼15 kb. Data are shown for both untreated cells (green line) and decitabine-treated cells (red line). (**C)** A heatmap representing the STAMP signal surrounding the TSS is shown for genes with a profile highly correlated to the composite density (A). Rows ordered by the location of mode and blue level is proportional to STAMP methylation signal. Each row represents an individual gene and columns represent distance from TSS as indicated. (**D**) A STAMP signal heatmap was generated for TTS as described in panel (C). Genes with a profile most similar to the composite density (B) are shown with rows ordered by STAMP signal. (**E**) Heatmap genes with poor correlation to the composite TSS density, as described for panel (B). (**F**) The correlation of each refGene to the composite TSS profile is plotted against the STAMP signal density (signal per bp) in the 1 kb surrounding the TSS. This plot demonstrates that refGenes with high signal near the TSS have a methylation pattern that is highly correlated to the composite profile shown in (A).

STAMP analysis revealed patterned DNA methylation at all scales across the genome: from individual genes (**Figs. 5** **,** **6**) to whole chromosomes (**Supporting Information S1)**. Much of what we understand about DNA methylation relates to transcriptional silencing associated with dense methylation of gene promoters but little is know about the role of methylation within intragenic regions such as exons. Because we found that 5′ and 3′ methylation represented two distinct classes (**Fig. 2**), we looked at the STAMP signal surrounding the transcription start site (TSS) and transcription termination site (TTS) of all 24,376 genes annotated in the UCSC refGene track. We identified a distinct central tendency in the STAMP methylation signal surrounding the TSS similar to that reported by Rauch et al and reminiscent of the overall pattern of CpG occurrence near TSSs [15], [33]. Genes with dense 5′ methylation dominate this profile and a more diffuse pattern emerges when these genes are excluded (**Supporting Information S1**). STAMP analysis exposed a previously unrecognized offset in the methylation peak which is ∼180 bp downstream from the TSS (**Fig. 5A**); quite close to the median length of first exons (209 bp). We initially suspected that this resulted from systematic inaccuracy in the refGene 5′ annotation due to the reverse transcriptase dissociating during cDNA production. However, the offset did not correct when we analyzed STAMP methylation surrounding 26,268 high-confidence TSSs annotated by SwitchGear Genomics (www.switchdb.com) (**Supporting Information S1**). Thus, dense methylation surrounding the TSS is maximal in the region of the first exon.

**Figure 6:**
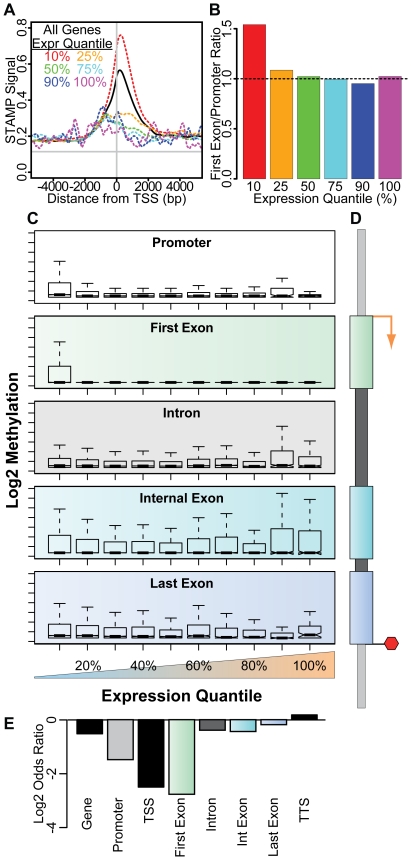
Correlation of transcript expression with the pattern of intragenic methylation in M091 cells. (**A**) The composite density plot of methylation surrounding the TSS is shown for all transcripts (solid black line) and for transcripts that are in various expression quantiles (dashed colored lines). Methylation for transcripts in the lowest 10% expression quantile (red) is significantly higher than for genes that are within the next 15% expression (orange). Transcripts with higher expression have even less methylation. (**B**) The ratio of first exon to promoter methylation is shown for transcripts in the lowest 10% (red), 10–25% (orange), 25–50% (green), 50–75% (cyan), 75–90% (blue) and 90–100% (magenta) expression quantiles. (**C**) Intragenic STAMP methylation is shown for transcripts in each of 10 expression quantiles (lowest 10% to 100%). Box plots of methylation within the promoter (white), first exon (green), any intron (grey), internal exons (cyan) and the last exon (blue) are shown as indicated. (**D**) Schematic of the promoter and intragenic elements is shown with the color code utilized in (C) and (E). (**E**) The odds ratio (log2 transformed), calculated using Fisher's exact test for count data, shows the likelihood of a transcript being expressed (greater than the lowest 10% expression quantile) if it is methylated (top 90% methylation quantiles) within the indicated component of the gene cassette.

To assess the methylation patterns of individual genes, we ranked each transcript by the similarity of its STAMP methylation profile to the composite TSS profile and identified a bivariate distribution in these correlations (**Supporting Information S1**). Genes with the highest correlation were predominantly those with the highest TSS methylation (**Fig. 5F**). To generate heatmaps, we selected genes with a correlation above (**Fig. 5C**) or below (**Fig. 5E**) a correlation breakpoint of 0.6. These heatmaps revealed distinct classes of methylation surrounding the TSS with local methylation being either concentrated just downstream of the TSS or unassociated with it.

We performed a similar analysis of the STAMP signal surrounding the refGene TTS (approximated as the 3′ end of the last exon) and found a pattern distinct from that at the TSS (**Fig. 5B**). In general, the STAMP signal gradually increases to a peak ∼940 bp upstream of the TTS and then drops to a minimum ∼220 bp downstream of the TTS (**Supporting Information S1**). To generate heatmaps, we ranked refGenes by their similarity to this composite profile near the TTS (**Fig. 5D** **and Supporting Information S1**). Unlike the distribution of TSS correlations, the distribution at the TTS was unimodal (**Supporting Information S1**). We again identified no correlation in the methylation at the 5′ and 3′ end of genes further suggesting that methylation within these regions is governed by distinct mechanisms (**Fig. 2** and **Supporting Information S1**). The composite DNA methylation patterns persisted when we treated the leukemia-derived cell line, M091, with decitabine (**Figs. 5A and 5B**) suggesting that decitabine reduced DNA methylation without preference for particular genomic positions and consistent with a dilutional model of hypomethylation.

### First exons have a distinct relationship to gene expression

Because the peak of DNA methylation was offset into the region containing the first exon, we next compared the pattern of methylation surrounding the TSS with the level of gene expression. We found that genes with the lowest expression quantile contain those with the highest level of 5′methylation. Genes with just 15% higher expression have vastly reduced 5′ methylation that is no longer offset from the TSS. This effect became even more pronounced for genes with higher transcripts levels (**Fig. 6A**). This analysis also demonstrates that, as a whole, genes with the lowest transcription have methylation that is shifted into the first exon region. Because 5′ methylation is skewed downstream from the TSS, we compared the level of first exon methylation to that within the promoter region for genes within each expression quantile. To do this, we compared the STAMP signal in equally sized regions either 250 bp upstream or downstream of the TSS for each refSeq transcript. We found that at least 45% more downstream (first exon) methylation compared to upstream (promoter) methylation in the lowest expressed genes (**Fig. 6B**, red bar). Genes with even modest expression showed no downstream methylation bias, again suggesting that methylation downstream of the TSS is tightly linked to transcriptional silencing (**Fig. 6B**). This result did not depend upon the size of the windows used surrounding the TSS (+/− 500 bp or +/−1000 bp), upon the cell type used (M091 cells or normal T cells) for analysis or whether we counted sequence tags in these windows instead of analyzing the STAMP signal. These results always demonstrated that methylation downstream of the TSS was always more closely linked to transcriptional silencing than methylation upstream of the TSS.

We then compared DNA methylation within individual elements of each gene cassette (i.e., promoter, first exon, introns, internal exons, and last exon) for genes within each of 10 expression quantiles (**Fig. 6C** **)**. These results pointed to a stringent requirement for hypomethylation of the first exon if the transcript is expressed. This requirement is more relaxed for the remainder of the gene cassette, including the promoter. We classified genes as either expressed (top 90% expression quantiles) or silenced (lowest 10% quantile) and as either methylated (top 90% methylation quantiles) or unmethylated (lowest 10% methylation quantile) to generate contingency tables for each component of the gene cassette. To quantify the strength of the correlations between expression and gene component methylation, we analyzed these tables using Fisher's exact test, as described previously (**Fig. 6E** **)**. Methylation of the first exon was the most strongly correlated with transcriptional silencing (log odds ratio, LOD, −2.8). Although there was also a clear negative correlation between expression and promoter methylation (LOD −1.5), this was not as pronounced as that seen for the first exon and within each expression quantile, we identified a number of genes with significant promoter methylation (**Fig. 6C** **)**. DNA methylation in the other regions of the gene body, including downstream exons, was only weakly linked to transcription level. So although first exon methylation is uncoupled from other gene body methylation, it is tightly linked to transcriptional silencing.

### Prominent first exon hypomethylation in transcripts upregulated by decitabine

**Figure 7:**
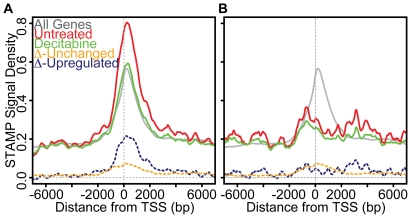
Decitabine induced hypomethylation of the first exonic region is associated with transcriptional activation. (**A**) Profiles of the composite methylation density surrounding the TSS of transcripts upregulated by decitabine are shown before (red) and after (green) decitabine treatment of M091 cells. For comparison, the composite methylation profile of all genes is shown as in Fig. 5A (grey). Also show is the change in methylation seen for transcripts that are upregulated (blue dashed line) or unchanged (orange dashed line) following decitabine treatment. (**B**) Profiles of the composite methylation density surrounding the TSS of transcripts downregulated by decitabine are shown before (red) and after (green) decitabine treatment. For comparison, the composite methylation profile of all genes is shown as in Fig. 5A (grey). Also show is the change in methylation seen for transcripts that are downregulated (blue dashed line) or unchanged (orange dashed line) following decitabine treatment.

Decitabine has proven useful in the treatment of several myeloid malignancies including AML. When we treated the AML cell line (M091) with decitabine, we identified ∼700 transcripts with modulated expression (**Supporting Information S1**). The vast majority of these were upregulated transcripts of genes involved in cell death, stress responses and differentiation. The smaller number of downregulated transcripts were predominantly genes involved in RNA processing and nucleic acid synthesis. Because transcriptional repression is closely linked to first exon methylation, we investigated how decitabine altered methylation at the 5′ end of genes that are induced or repressed by decitabine (**Fig. 7**). Looking at DNA methylation prior to and after treatment, we found that hypomethylation is strongly biased towards the first exonic region in genes that are induced following decitabine treatment (**Fig. 7A**). This bias is far more pronounced than that seen at the 5′ end of genes with negligible changes in expression level. In contrast, genes downregulated by decitabine had little 5′ methylation and the methylation present was skewed away from the first exon (**Fig. 7B**). These downregulated genes likely represent secondary targets that are repressed as a consequence of decitabine treatment rather than from a change in their DNA methylation.

## Discussion

Although transcriptional repression is associated with promoter methylation, we found that it is more assured with methylation of the first exon. Our studies represent the first detailed analyses of regional gene body methylation and its relationship to transcript expression. We found that most dense genomic methylation occurs outside of classical CGIs. These DMEs are preferentially located within gene bodies with a bias for exonic regions. Although gene body methylation is common, we found that the relationship between DNA methylation and expression is complex and closely linked to the intragenic location of the methylated elements. Strikingly, we found DNA methylation downstream of the TSS is the most critical for transcriptional silencing.

Exciting new technologies have both expanded our understanding of genomic methylation and opened new controversies [1], [3], [18], [22], [35], [36], [37], [38], [39], [40], [41], [42]. It is now evident that most of the methylated human genome lies outside the context of CGIs. Much of this methylation is constitutive and occurs in regions of low CpG density [32], [37], [43]. In contrast, tissue specific methylation generally occurs in regions with higher CpG content, although not necessarily in CGIs. We found that the vast majority of DMEs do not overlap classical CGIs (**Fig. 2A**) but those that do, generally do so completely with the DME extending beyond the flanks of the CGI into regions conceived as CGI “shores” [44]. The definition of CGI is based upon sequence characteristics and relatively arbitrary cutoffs. Efforts to objectify the definition of CGI have been reported but have not been widely adopted [14], [45]. We identified DMEs using a functional assay and found that the sequence characteristics of DMEs are distinct from CGIs and from the bulk of genomic CpG dinucleotides. Interestingly, although tissue specific patterns of DMEs are clearly evident, their sequence characteristics do not vary much suggesting that DMEs are drawn from a larger cohort of *potentially* methylated elements (Scandura, unpublished). This is important because it is a subset of these potentially methylated regions that undergo tissue specific methylation. Our results will be useful for the functional validation of new CGI definitions.

Transcribed genes have extensive DNA methylation throughout their bodies yet the relationship between this methylation and transcription is controversial [4], [18], [33]. Two factors appear to be responsible for the discrepancies: the use of diverse technologies with different sensitivities to DNA methylation density; and analytical approaches that couple composite methylation measures to gene expression. Owing to the sensitivity of STAMP to methylation density (**Supporting Information S1**), our analysis adds to these prior reports by isolating the contribution of dense regional methylation from the low-density constitutive methylation that predominates in gene bodies. We found that composite gene body methylation (i.e., all methylation between the TSS and TTS) affected transcription only modestly (**Fig. 6**) whereas methylation of low-density regions is reported to track with expression [18]. These results suggest that the cellular interpretation of regional methylation depends upon whether it is dense or sparse [23], [24], [25], [26]. Yet, composite measures of gene body methylation do not account for the biological non-equivalence of intronic and exonic DNA. So while constitutive, low-density methylation may guard against the initiation of spurious transcripts that can cause polymerase collisions, the function of dense intragenic methylation may depend upon where the methylation occurs.

We found that ∼20% of all internal exons have dense methylation across their entire span. This contrasts with methylation within intronic regions that generally encompasses a small portion of the intron length (**Figs. 2E–F** **&** **3**). Although downstream exonic methylation has been reported previously, our results demonstrate that it is a widespread phenomenon, albeit one with no assigned function. Jones originally noted the “paradox” that methylation of downstream CGIs does not block transcription initiated upstream and proposed that transcription through a CGI facilitates *de novo* methylation [46]. Our results argue that this relationship must be more complex. We found that dense downstream methylation had a weak negative association with the amplitude of transcription arguing against a transcriptional trigger for this methylation (**Fig. 6E**). Furthermore, we found that the methylation of internal exons was highly selective with methylated exons generally surrounded by exons with no methylation. The exon chosen for methylation was not predicted by its CpG dinucleotide content suggesting that the preference for a particular exon is biological. Our results invite the discovery of a function for downstream exonic methylation and strongly suggest that those looking to solve this enigma seek a mechanism that is uncoupled from regulation of transcriptional magnitude.

The association of promoter methylation with transcriptional silencing is well recognized and certainly our data demonstrate the same. Yet, we found that methylation downstream of the TSS, in the region of the first exon, is much more tightly correlated with transcriptional silencing than is methylation upstream of the TSS, in the promoter region. Prior elegant studies by Okitsu and Hsieh also showed that methylation in the region of transcript initiation/elongation is most important for transcriptional suppression, at least in the context of “patch” methylated stable episomes [47]. Our results demonstrate that this observation can be generalized. By blocking transcription initiation or causing proximal polymerase pausing [48], DNA methylation of the leading exon can block effective transcription whereas methylation of downstream exons can still permit the passage of transcripts initiated upstream. Such a model allows first exon methylation to govern the selection of alternative starts.

Strikingly, we found that methylation was even excluded from the first exon of genes with very low-level expression. Because most genomic CpG dinucleotides are methylated, these results tacitly require a biological means with which to prevent first exonic methylation. One possibility is that epigenetic configurations that support transcription inhibit those promoting DNA methylation. Indeed, tri-methylation of histone H3 lysine 4 (H3K4me3), a mark localized to the proximal regions of genes poised for transcription [49], [50], is inversely correlated with DNA methylation [32]. Similarly, RNA polymerase II localized near the TSS in normal mammary or prostate epithelial cells predicts genes that are unlikely to be methylated in prostate or breast cancers [51]. Our data suggest that transcript initiation may play a pivotal role in protecting the first exon from encroaching methylation.

Aberrant DNA methylation is a common means by which tumor suppressor genes (TSGs) are inactivated during carcinogenesis [52], [53], [54]. Unlike genetic mechanisms of gene inactivation, such as gene deletion and mutation, the epigenetic silencing of TSGs by DNA methylation is potentially reversible. This has led to the broad interest of cancer biologists in the study of DNA methylation. We analyzed expression and methylation patterns in AML cells before and after treatment with decitabine. Despite its broad hypomethylating activity, decitabine regulated a modest number of genes suggesting that hypomethylation of specific loci in particular cellular contexts is required to affect transcription. The majority of the genes were upregulated and, as a whole, these showed disproportionate hypomethylation of the 5′ end with a preference for hypomethylation of the first exon. This was not seen for downregulated transcripts and was greatly attenuated for genes with insignificant changes in expression. These results further support the notion that first exonic methylation is linked to transcriptional silencing and argues against a general linkage between composite gene body methylation and transcription.

The 4N nature of the bisulfite genome makes large-scale bisulfite sequencing projects both resource and computation intensive [4], [21], [55]. Recent reports demonstrate that even after several billion fragments are sequenced, almost a quarter of the human bisulfite genome is represented by just a few sequence traces, and more than a third of all CpG dinucleotides are unanalyzed [4]. STAMP analyzes a reduced complexity genome to robustly identify methylated DNA segments with just a few million mapped reads per specimen. However, this efficiency comes with a restricted ability to discern methylation in regions with sparse CpGs (**Supporting Information S1**), as reported for similar technologies [38], [39], [56]. STAMP does not require high molecular weight DNA, and does not suffer from sequence bias introduced by direct linkage to particular restriction sites or from fragment length-dependent amplification effects. The non-restrictive DNA requirements and cost effectiveness of the STAMP method make it an approachable alternative to genome-wide bisulfite sequencing. This technique permits even small labs to routinely perform genome-wide analyses of DNA methylation to identify biologically and medically relevant patterns.

A recent explosion of data has exposed both the breadth of genomic DNA methylation and our limited understanding of its significance. We found that dense exonic methylation occurs far more frequently than previously recognized. But the manner with which exonic methylation relates to transcription is linked to the relative position of the methylated exon. Only first exonic methylation is tightly associated with transcriptional silencing. Our data make it clear that the transcriptional apparatus perceives methylation of more downstream exons distinctly. It is tantalizing to suggest that such methylation may help guide the alternative splicing that is seen in almost half of all protein coding genes [57]. Although the functional assignation of all genomic methylation awaits further exploration, our data suggest that we must now begin thinking about functions of DNA methylation that extend beyond simple associations with overall transcript level.

*Note in added proof*: Following our submission two additional surveys of genome-wide DNA methylation have been published demonstrating widespread intragenic methylation and a preference for coding regions such as exons [58], [59].

## Methods

### His-MBD production

A fragment of MBD1 coding for amino acids 1 to 69 was amplified by PCR from human cDNA synthesized from M091 total RNA. The PCR fragment was cloned into pENTR/D-TOPO plasmid and propagated in TOP10 bacteria (Invitrogen). The insert was fully sequenced and then recombined into the pDEST-17 bacterial expression vector using the Gateway system (Invitrogen). Recombinant His-MBD protein was purified from inclusion bodies of 500 ml BL21-AI cells 24 hours after induction with 0.2% L-arabinose. Inclusion bodies were sonicated briefly and washed in 1 M Urea, 20 mM Tris-Cl pH 8, 10 mM β-mercaptoethanol, 2% Triton X-100 prior to solubilization in Denaturation Buffer (8 M Urea, 20 mM Tris pH 8, 5 mM β-mercaptoethanol). Partially purified, denatured recombinant His-MBD was purified to homogeneity (by SDS-PAGE) on Ni-NTA-agarose beads (Qiagen). Protein was refolded by rapid dilution into MBD Refolding Buffer (20 mM HEPES pH 7.4, 150 mM NaCl, 0.1% Tween-20, 10 mM β-mercaptoethanol) to achieve a final dilution of 24-fold and a final protein concentration of ≤50 µg/ml. Refolded protein consistently demonstrated high selectivity for methyl-CpGs with no detectable binding to unmethylated CpGs (**Supporting Information S1**).

### DNA purification and fragmentation

The human acute myelogenous leukemia-derived cell line, M091, was propagated in RPMI 1640 as described [27]. Prior to harvest, cells were plated in replicate cultures at a density of 10^5^/mL and grown either in the absence (untreated) or presence of 5-aza-2′-deoxycytidine (decitabine) for three days. Decitabine (Sigma) was added to a final concentration of 1 µM every 24 hours. Viability of MO91 cells was not altered by a three day treatment with decitabine although longer exposure (5, 7 and 10 days) caused progressive cell death. Primary human T cells and granulocytes were purified from the blood of healthy donors following written informed consent. All donor consent forms and specimen utilization procedures were approved by the Weill-Cornell Medical College Institutional Review Board. We prepared genomic DNA from the cells by overnight Proteinase K treatment, RNAse-digestion, phenol-chloroform extraction and ethanol precipitation. Purified genomic DNA was fragmented by sonication (Misonix 3000) to a modal size of ∼200 bp. In subsequent work, we have fragmented DNA using acoustically focused sonic disruption to achieve a modal fragment length of ∼110 bp and a tight distribution of fragment lengths. The use of shorter DNA fragments is advantageous for specimen processing but does not affect the distribution of MBD-enriched sequence tags.

### STAMP assay library preparation

Refolded His-MBD protein (10 µG) was collected on 150 µl prewashed Dynal Talon beads (Invitrogen) in MBD Refolding Buffer by rotation at 4°C for 30 min. Beads were washed 3 times with 500 µl MBD Refolding Buffer and then another three times with 500 µl MBD-Talon Buffer (10 mM Tris pH 7, 140 mM NaCl, 0.05% Triton X-100, 0.5% BSA) before being resuspended in 100 µl of MBD-Talon Buffer. For enrichment of methylated DNA, 1 µg randomly fragmented DNA in TE (10 mM Tris pH 8, 1 mM EDTA) was adjusted to a volume of 200 µl TE before addition of 100 µl 3X MBD-Talon Buffer. To this, 20 µl washed MBD-Talon beads (2 µG His-MBD) were added and the mixture was rotated overnight at 4°C. Beads were subsequently washed 3 times with MBD-Talon Buffer and then resuspended in 100 µl Elution Buffer (1% SDS, 10 mM EDTA. 50 mM Tris pH 8) containing 50 µg Proteinase K (Sigma). After incubation at 55°C for 1 h, DNA was purified by phenol-chloroform extraction and ethanol precipitation. MBD enriched fragment libraries were prepared using a modification to the SOLiD genomic DNA sample preparation protocol. Briefly, DNA ends were polished using the End-It kit (Epicentre) and then purified using MinElute reaction cleanup column (Qiagen). The DNA was then ligated to the SOLiD A/B linkers and purified per the standard protocol. The DNA was then pre-amplified for 8 cycles.

### SOLID sequencing and computational methods

Emulsion PCR and sequencing was performed using the standard SOLiD 2 system for 35 bp reads. Raw color-space data was mapped to the human genome (hg18) using corona-light (ABI). The sequence tag start and strand was imported into a custom R-language data structure for analysis. To correct for minor differences in the sequencing depth between specimens, the total number of tags was normalized to 10^6^ for each specimen by dividing each tag weight by the total number of tags and multiplying this by 10^6^. To calculate the STAMP signal, each tag was extended to a distribution of lengths modelling the DNA fragmentation pattern (**Supporting Information S1**). It is necessary to track Watson and Crick DNA mapped strands to determine the direction in which the mapped end should be extended during STAMP analysis. These tag densities were then summed to generate a methylation signal (**Fig. 2**). We used this approach to calculate a STAMP signal surrounding all TSS, TTS, at CpGs interrogated by the Illumina HumanMethylation27 microarray and for 15,000 randomly selected genomic loci. The composite methylation profiles at the TSS and TTS are determined by the superposition of all enriched fragments mapping near the TSS. Individual fragments contribute little to this compound signal and the profiles are insensitive to the fragmentation profile of the DNA.

To assess noise, we calculated a composite STAMP signal from the sequence tags within random 15 kb windows for all samples. We defined the mean STAMP signal density within these windows as the noise floor (NF). Using this approach, we found that NF was uniformly 0.114. The NF estimate was independent of the sample and is approximately equal to one sequence tag per kb when the total number of sequence tags per data set is scaled to 10^6^.

To identify DMEs, we first chose all regions with STAMP signal greater than a threshold value. Then the flanks of those regions were extended until the signal declined to 4× NF. To minimize false discovery of DMEs, the detection threshold value was chosen to ensure that the number of DMEs identified in unenriched DNA was less than 5% of that identified in an His-MBD enriched sample from the same source (**Supporting Information S1**).

Comparison of DNA methylation across genomic regions with varying CG density was performed by classifying each region as either methylated or unmethylated (lowest 10% of sequence tags for regions with similar sequence content) prior to the generation of contingency tables. Fisher's exact test for count data was used to assess the likelihood of any genic element being methylated if any other element is methylated. This approach largely uncouples the analysis from CG density because the element class assignment is insensitive to the magnitude of the STAMP signal. We also calculated the CpG density (CG_f_), GC fraction (GC_f_) and CG_oe_ ratio (CG_oe_ = CG_f_/(G_f_ * C_f_) in sliding windows of 200 bp tiled every 10 bp across the entire human genome. We then identified the fraction of each genic element that could be classified as HCP, ICP or LCP as defined by Weber et al [37], and the fraction of the element detectable (CGf>0.1) by STAMP. Analyses performed using subsets of the genome restricted by these various sequence classes had minimal effect on the results and did not alter the interpretation. Data presented in Figs. 2 and 5 was analysed for the STAMP detectable portion of each genic element.

To generate density plots of CGoe vs GC fraction (**Fig. 4**), we first analyzed the sequence characteristics of a 200 bp window surrounding each of the ∼28 million CGs in the human genome. We then performed similar analyses for each CG within an annotated CGI and within each of the DMEs we identified. This analysis was performed using custom written tools written in R, utilizing Bioconductor packages BSgenome and IRanges [60].

### Bisulfite DNA analysis

A portion of the genomic DNA extracted from the same cells as analyzed by STAMP was bisulfite converted (Zymo Research Corp.) prior to fragmentation. Bisulfite-treated DNA was analysed at selected genomic loci by quantitative PCR, Methylight [28], by deep sequencing using 454 Titanium Sequencer and by using the Illumina HumanMethylation27 microarray. Illumina arrays were processed as per manufacturer's instructions and data was extracted using BeadStudio software. Deep bisulfite amplicon sequencing was performed using standard 454 emulsion PCR processing. Sequenced amplicons (**Supporting Information S1**) were mapped to both the Watson and Crick bisulfite genome. Fractional CpG methylation was calculated at each CG dinucleotide mapped to the amplicon locus as f_mCpG_ = N_Ci_/(N_Ti_+N_Ci_), where N_Ti_ and N_Ci_ are the number of reads with a C or T in position i.

### Gene Expression Analysis

Total RNA was extracted from cells with Trizol using standard procedures and RNA quality was assessed using a BioAnalyzer (Agilent). RNA was labelled and hybridized to Illumina Human Ref8 microarrays as per manufacturer's instructions and data was extracted using BeadStudio software. All subsequent analysis was performed in the R programming environment utilizing Bioconductor packages and custom procedures. Preprocessing of raw data was performed using the lumi package. Differentially expressed transcripts were identified after empirical Bayesian modelling and analysis using the limma package. Gene ontologies overrepresented within the differentially expressed transcripts were identified after calculating hypergeometric p-values conditionally using the structure of the gene ontology database within the GOstats package.

## Supporting Information

Supporting Information S1 Supporting tables, figures and methods.
(19.46 MB PDF)
