# Supplementary material for: DNA Methylation of the First Exon Is Tightly Linked to Transcriptional Silencing
Source: PLoS One. 2011 Jan 18;6(1):e14524. doi: 10.1371/journal.pone.0014524 (PMC3022582; doi:10.1371/journal.pone.0014524)
Supplement: Supporting Information S1 — Supporting tables, figures and methods. (19.46 MB PDF) [file pone.0014524.s001.pdf]

SUPPORTING INFORMATION

Supporting Table 1: Description of the primers and probes used in Methylight analysis.
(Methods and Results: Genome-wide identification of densely methylated elements)

Supporting Table 2: Amplimers used for deep bisulfite sequencing.
(Methods and Results: Genome-wide identification of densely methylated elements)

Supporting Figure S1: His-MBD binding to methylated and unmethylated oligonucleotides.
(Methods and Results: Genome-wide identification of densely methylated elements)

Supporting Figure S2: Schematic describing STAMP signal calculation and DME identification.
(Methods and Results: Genome-wide identification of densely methylated elements)

Supporting Figure S3: Bisulfite sequencing validation of STAMP signal at the CDKN2B locus.
(Results: Genome-wide identification of densely methylated elements)

Supporting Figure S4: Specimen discrimination and reproducibility of STAMP signal.
(Results: STAMP assay is highly reproducible and sensitive to methylated CpG density)

Supporting Figure S5: Comparison of STAMP signal to 27,578 CpG interrogated by the Illumina HumanMethylation27 Infinium BeadChip array.
(Results: STAMP assay is highly reproducible and sensitive to methylated CpG density)

Supporting Figure S6: "Macroscopic" chromosomal STAMP patterns.
(Results: Patterned DNA methylation at the 5' and 3' ends of genes)

Supporting Figure S7: STAMP signal patterns for genes with the highest or lowest detectable methylation surrounding the TSS and TTS of all refGenes.
(Results: Patterned DNA methylation at the 5' and 3' ends of genes)

Supporting Figure S8: STAMP methylation offset at the TSS and the TTS.
(Results: Patterned DNA methylation at the 5' and 3' ends of genes)

Supporting Figure S9: DNA methylation patterns at the TSS and TTS.
(Results: Patterned DNA methylation at the 5' and 3' ends of genes)

Supporting Figure S10: Gene ontology of decitabine-regulated transcripts.
(Results: Prominent first exon hypomethylation in transcripts upregulated by decitabine)

Supporting References

Name	Primers Sequence 5' to 3'
LINE-1 Methylight	GTT AGA TAG TGG GCG TAG GTT ATT G (F)<br>TTA CGC TTC CCA AAT AAA ACA ATA CC (R)
CDKN2B Methylight	CGC CCG CTA ATT CTT AAA CGC (F)<br>GTT TGG GGT TTC GTG TAG TGG (R)
GAPDH Methylight	ACC TTA AAC TAAAAC TAA CTA AAC CTA ACG (F)<br>GTT GAT TGT CGA ATA GGA GGA GTA G (R)
ALU Methylight*	GGT TAG GTA TAG TGG TTT ATA TTT GTA ATT TTA GTA (F)<br>ATT AAC TAA ACT AAT CTT AAA CTC CTA ACC TCA(R)
**Methylight Probe Sequence 5' to 3' [a]**	
LINE-1	CCT ACT TCG ACT CGC GCA CGA TAC G-IBFQ[c]
CDKN2B	ACC CGA AAC TAA CGA CCG ACC GCT C
GAPDH	AAT CCG AAT CAC CGC CTA CCG CCG
ALU	CCT ACC TTA ACC TCC C-MGB[b]

Supporting Table 1: Description of the primers and probes used in Methylight analysis.[a] All probes contain a 6FAM fluorophore and a BHQ-1 probe unless otherwise noted. [b] MGB refers to a Minor Groove Binder non-fluorescent quencher in the 3' terminus of the probe. [c] IB FQ refers to an Iowa Black Fluorescence Quencher in the 3' terminus of the probe. * See Weisenberger et al., 2005[1]

Primer Name	Sequence (5' to 3')	Start	Product Length (bp)
CDKN2B-BS-000_10F	TTTGTATTGAGTTTGTAATAGTGTTATTG	21994919	
CDKN2B-BS-000_T7R	CAACTCCACCAAATAACAAAAAAAT	21995364	445
CDKN2B-BS-001_10F	TTTTATTTTTTTGTTATTTGGTGGA	21995336	
CDKN2B-BS-001_T7R	AAAAATACATATAAAATCCTTAACTAAACC	21995687	351
CDKN2B-BS-002_10F	TTAGGGTTTAAGTTGTGGGTTTATT	21995511	
CDKN2B-BS-002_T7R	CAAACCTATCTAAAACTCACAAAAA	21995871	360
CDKN2B-BS-003_10F	TTTTGTGAGTTTTAGATAGGTTTGTAGG	21995848	
CDKN2B-BS-003_T7R	TAAATAACTCCACCTACCTTACCCC	21996332	484
CDKN2B-BS-004_10F	TAGGTTTTTGATGTTTGGTGTTTTT	21996377	
CDKN2B-BS-004_T7R	TCACACTACAAACCAATTATAAACTCA	21996849	472
CDKN2B-BS-005_10F	AGAGTTTGGGTTAGGTTTAGTGGTT	21997070	
CDKN2B-BS-005_T7R	ACTACAAAATTACTCCTACAAAACATCA	21997466	396
CDKN2B-BS-006_10F	TTTTTTTTAGGATTTTTGTTGGGTA	21998621	
CDKN2B-BS-006_T7R	AAAATAATAAAACTAAACCCAAATCTCC	21999090	469
CDKN2B-BS-007_10F	TTTTTTTTAGGAGATTTGGGTTTAG	21999054	
CDKN2B-BS-007_T7R	TCCTAACATCTTTAAACAAACTTCCC	21999464	410
CDKN2B-BS-008_10F	TTTGGGAATGTTTATTATTGTTTTT	21999551	
CDKN2B-BS-008_T7R	ATACCAAATTACCACTCTCAATCTC	21999979	428
CDKN2B-BS-009_10F	AATTTATTTGGATAGGGAAGGGAAT	22000666	
CDKN2B-BS-009_T7R	AAAACTAATTAAAACCACCCAAACC	22001157	491
CDKN2B-BS-010_10F	GAAGTAAGTTGATTGAATGAAAAATAAT	22001033	
CDKN2B-BS-010_T7R	AATCACTCAAATTCTCTAATCCTTTTAAC	22001492	459
CDKN2B-BS-011_10F	TGGGAATATAGTGGGTAGTTGAAAT	22001853	
CDKN2B-BS-011_T7R	TCAAAAAAAATACCCTCCTTATCTT	22002270	417
CDKN2B-BS-012_10F	TTTGTTAGTAAATAGTTGGAGGATGG	22002287	
CDKN2B-BS-012_T7R	AACCCCAAAAATAATTATAAATTTCC	22002773	486
CDKN2B-BS-013_10F	GAATTGGTTGATTGGTTAAGAGAAA	22002642	
CDKN2B-BS-013_T7R	TAAACACACACAAAAAATCCAAAAA	22003129	487

Supporting Table 2: Amplimers used for deep bisulfite sequencing. Primers span the CDKN2B locus. The hg18 genomic positions on chromosome 9 are listed (Start).

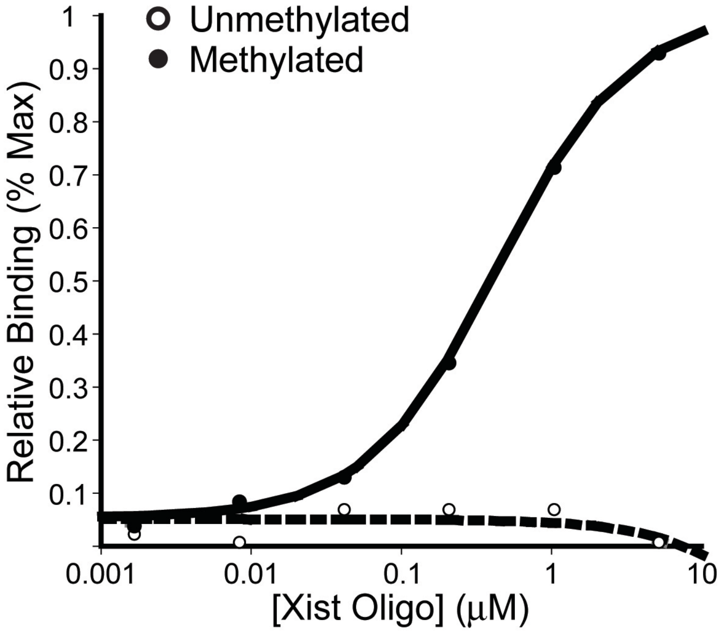

Supporting Figure S1: His-MBD binding to methylated and unmethylated oligonucleotides. His-MBD was mixed with the indicated concentration of biotinylated oligonucleotides with either two (Methylated) or no (Unmethylated) methyl-CpG dinucleotides. The sequence of the oligonucleotides is from a region of the human Xist promoter. His-MBD was collected on Talon beads and washed three times with buffer. The amount of oligonucleotide bound to His-MBD was assessed by incubation of the beads with streptavidin-HRP followed by three additional washes. The amount of HRP was measured in a spectrophotometer using the colorimetric substrate TMB (3,3´,5,5´-tetramethylbenzidine) and normalized with a standard curve generated with known concentrations of HRP.

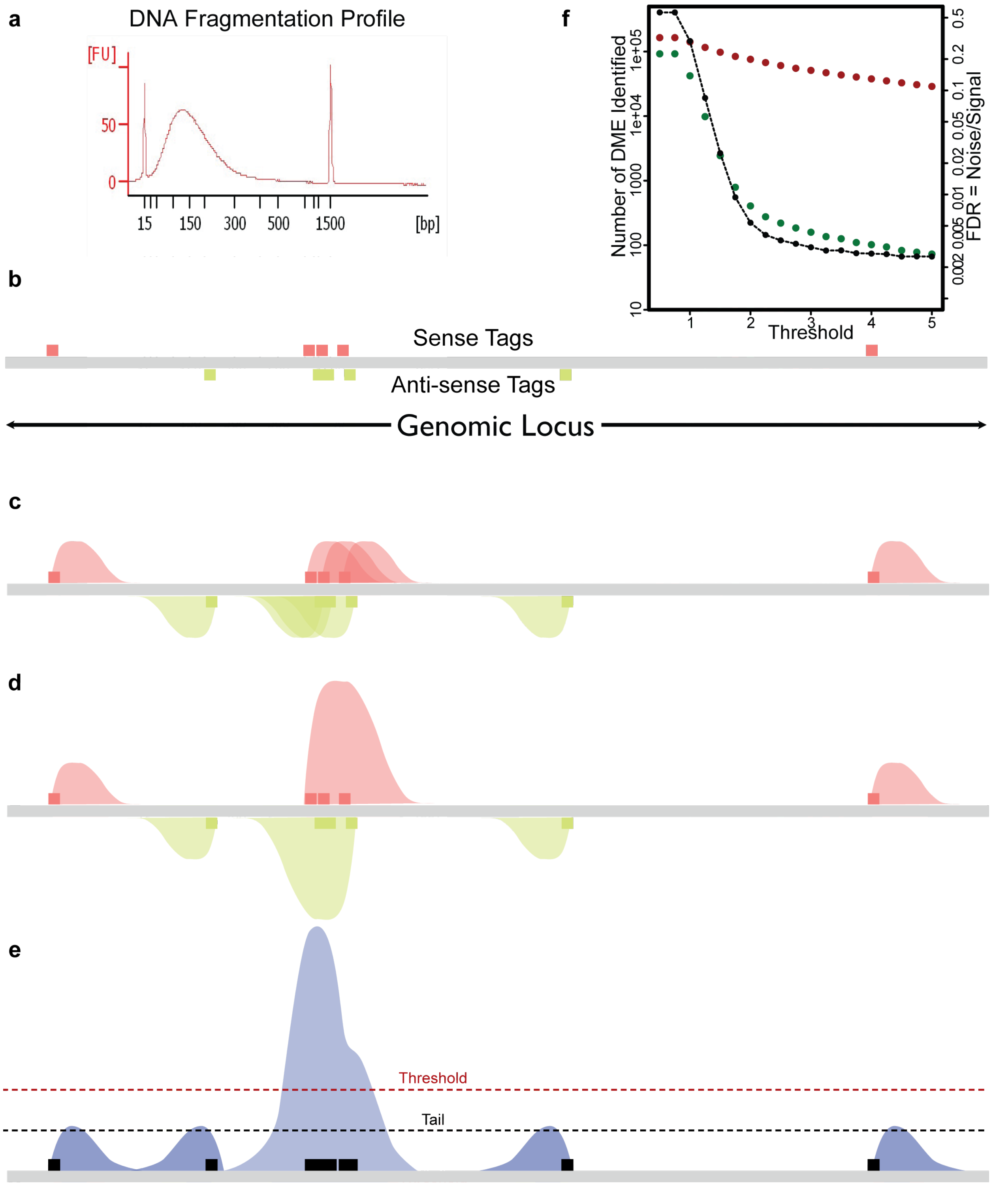

Supporting Figure S2: Schematic describing STAMP signal calculation and DMR identification. Each sequence tag is extended to a distribution of lengths modeled by the fragmentation pattern (**a**). (**b**) Initially, each tag is mapped to the genome. Each tag represents a DNA fragment of an unknown length picked from the distribution of lengths. (**c**) This is modeled by spreading the density of each tag across a local genomic region. These convolved tags are then summed across both strands (**d**) to yield a signal (**e**). DMEs are identified as regions with a maximum signal above a threshold and a span that extends to a second threshold (Tail). (**f**) To ensure low false discovery, the DME threshold is selected to minimize the ratio of the number of DMEs identified (left axis) in the MBD-enriched DNA (signal, red) compared to those identified in a matched unenriched specimen (noise, green). The black symbols show the FDR (right axis) for each threshold selected. A threshold of 2 was selected for analysis of DMEs.

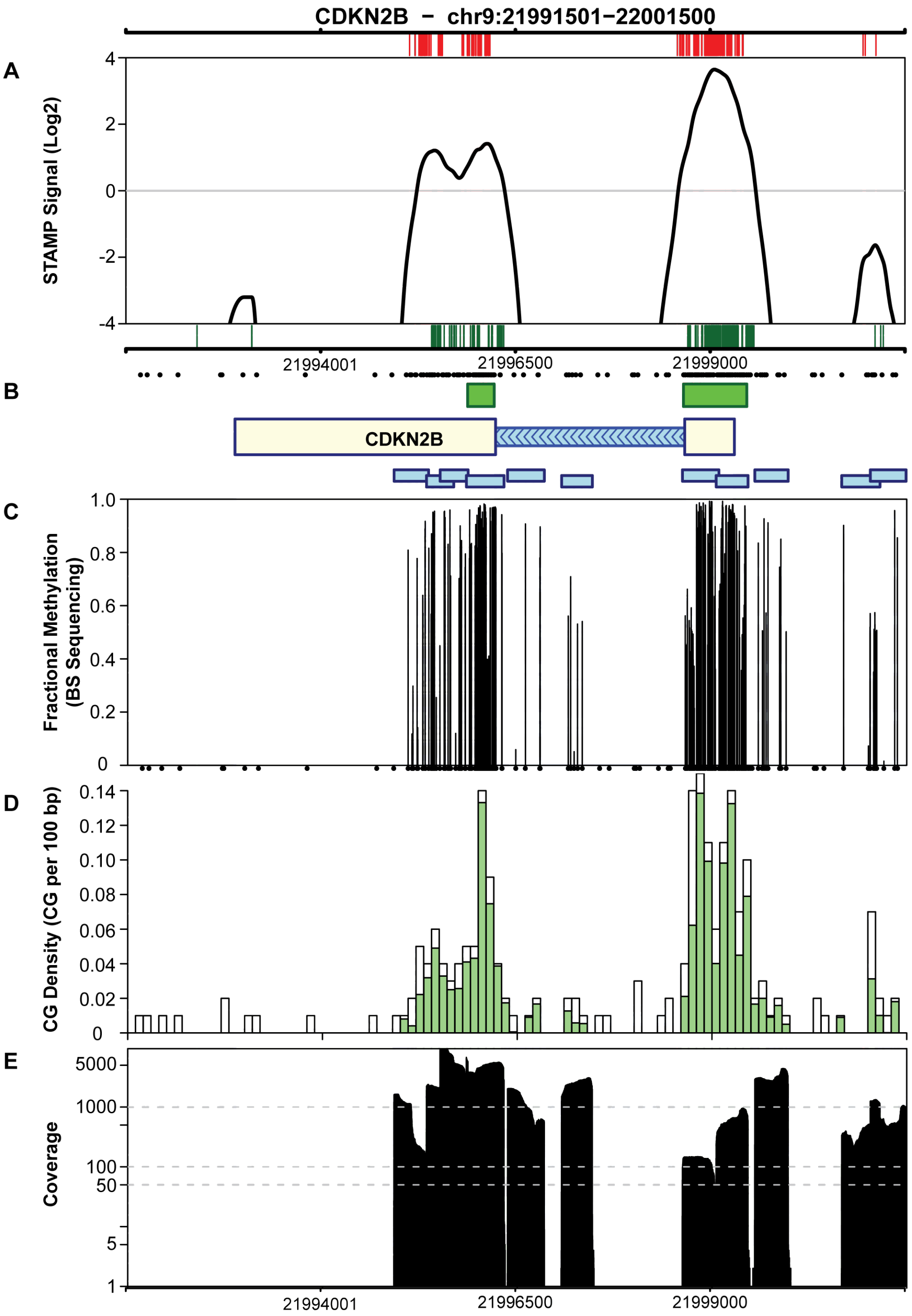

Supporting Figure S3: Bisulfite sequencing validation of STAMP signal at CDKN2B locus. (**A**) Sequence tag maps and methylation profile is shown at the CDKN2B locus for the AML cell line M091. Upper vertical red bars and lower vertical green bars represent individual sequence tags mapping to the sense and antisense strands, respectively. The black line represents the composite STAMP signal (log2). (**B**) A schematic of the gene locus including the location of CG dinucleotides (black dots), CpG Islands (green boxes), the gene body (exons pale yellow, introns light blue with direction of transcription shown by inset arrow heads) and the location of bisulfite sequencing amplicons (blue boxes) are shown top to bottom, respectively. (**C**) The fraction of CG methylation detected by direct bisulfite sequencing is shown for all sequenced amplicons. Sequencing was performed using 454 Titanium chemistry. (**D**) The CG density within 100 bp bins is shown across the locus (white boxes). The density of methylated-CG detected by bisulfite sequencing is shown in pale green. (**E**) The sequence coverage is shown and ranges from ~200x to 6000x for each amplicon.

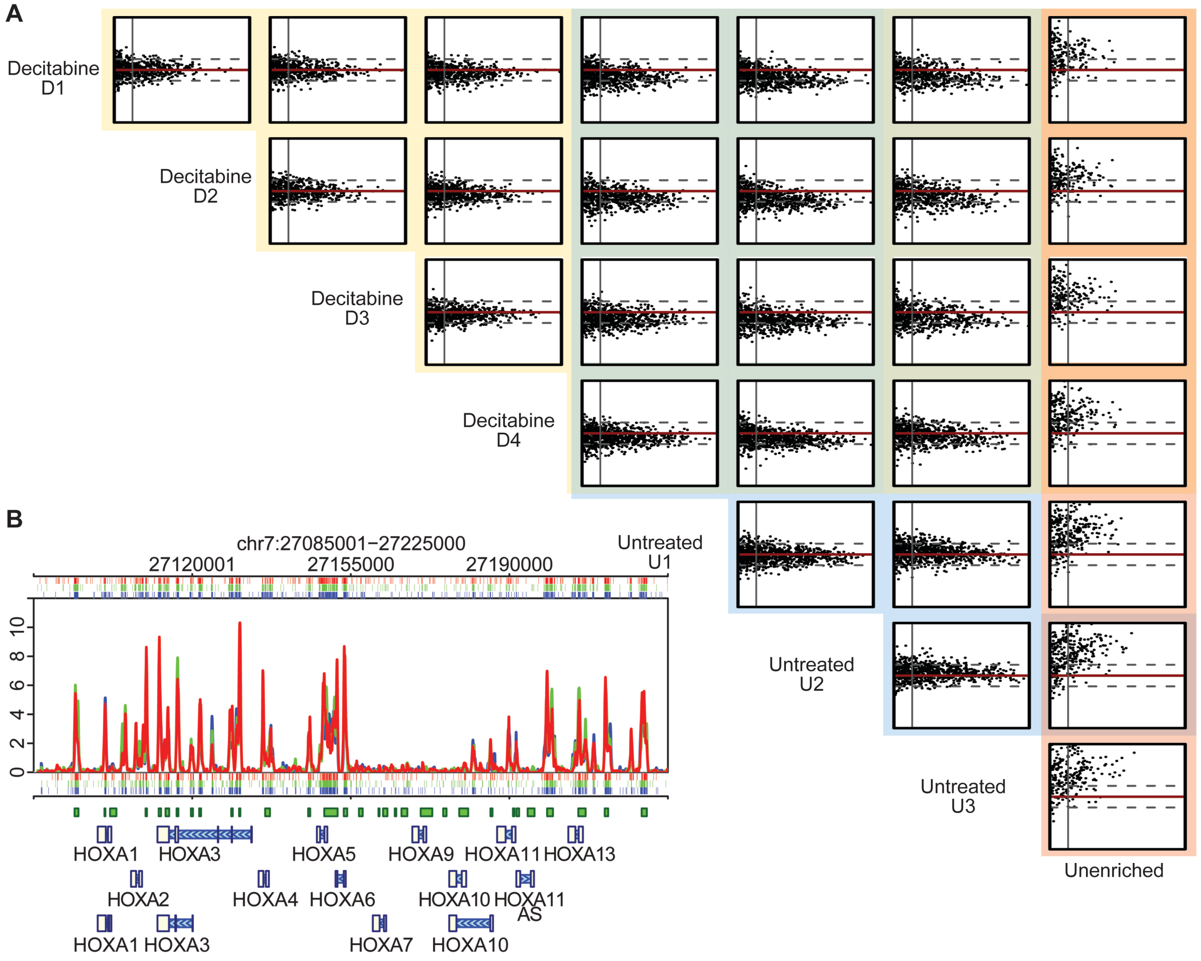

Supporting Figure S4: Specimen discrimination and reproducibility of STAMP signal. (**A**) Methylation signal of decitabine treated and untreated biological replicate specimens of M091 cells were compared at 15,000 randomly selected genomic loci. For each pair of samples, the ratio of the log2 STAMP signal (y-axis) was plotted against the average log2 signal (x-axis) at each genomic locus (MA plot). The horizontal red line represents a signal ratio of 1. The horizontal grey dashed lines represent two-fold changes in the signal ratio. Little deviation from a unitary signal ratio is seen in biological replicates of untreated and decitabine-treated cells, yellow and blue background, respectively. In contrast, the decitabine-treated cells have a systematically decreased STAMP signal when compared to untreated cells (green background). All of the His-MBD enriched data has greater signal and no correlation to the unenriched DNA (purple and orange boxes). (**B**) The STAMP signal is plotted at the HOXA locus for three independent biological replicates to illustrate the highly reproducible nature of the methylation pattern. The clustered Hox loci are particularly informative because their regional activation yields striking epigenetic pattern with highly methylated regions adjacent to genes with little detectable methylation.

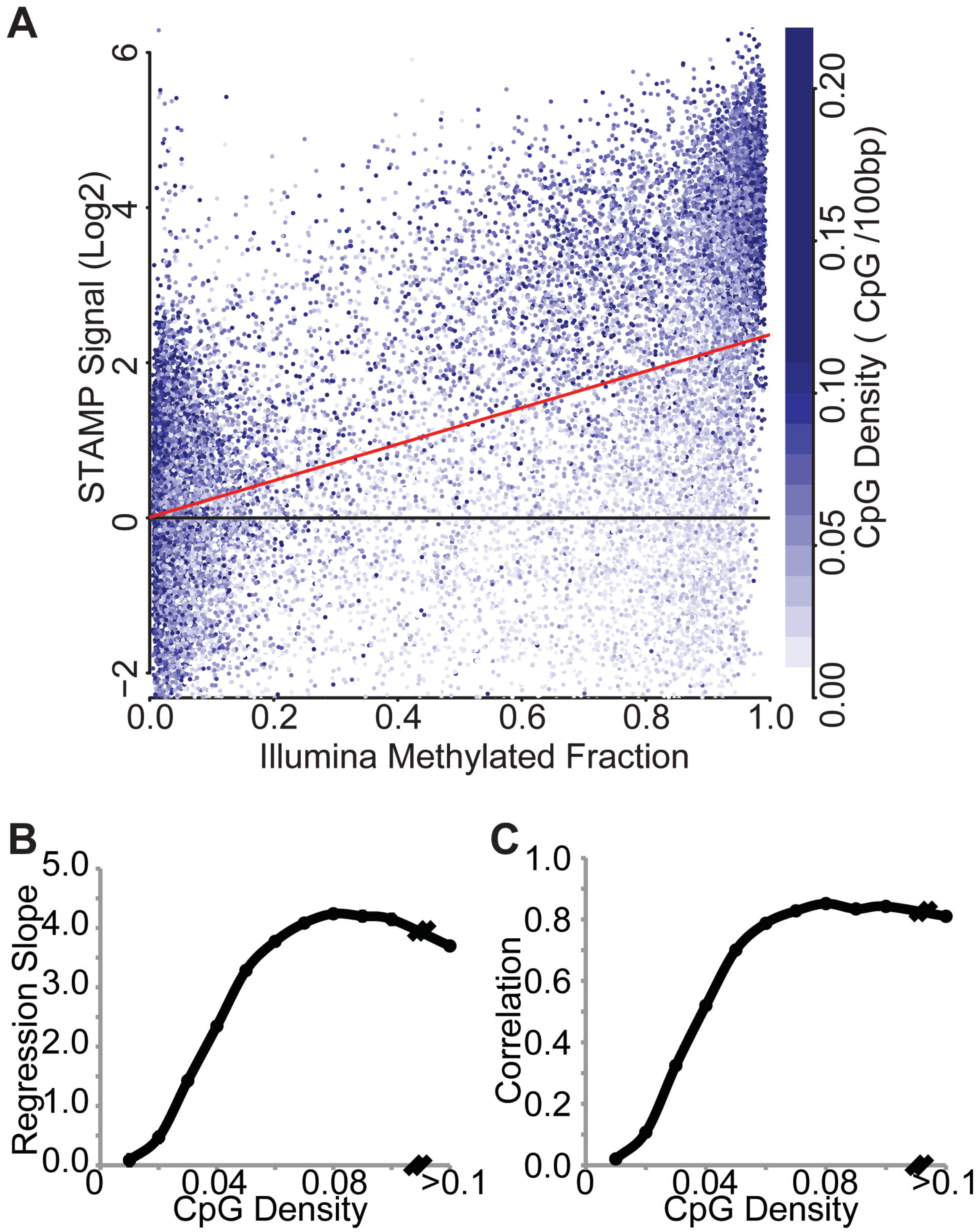

Supporting Figure S5: Comparison of STAMP signal to 27,578 CpG interrogated by the Illumina HumanMethylation27 Infinium BeadChip array. (**A**) The x-axis is the mean fractional methylation (average beta) reported on the Illumina array: signal ranges from 0 to 1. The y-axis is log2 of the computed STAMP signal at the precise nucleotide interrogated on the Illumina array. Each location (point) is colored by the density of CpG dinucleotides in the 100 bp surrounding the nucleotide probed by the Illumina array. A log-linear relationship between the data sets is shown by a regression line with an overall correlation=0.50, log2STAMP = 2.4*fractional methylation (red line). (**B & C**) Similar regression lines were calculated for probe bins having different local CpG densities. A log-linear relationship was maintained but the slope of the regression (**B**) and the correlation between data sets (**C**) varied as a function of CpG density.

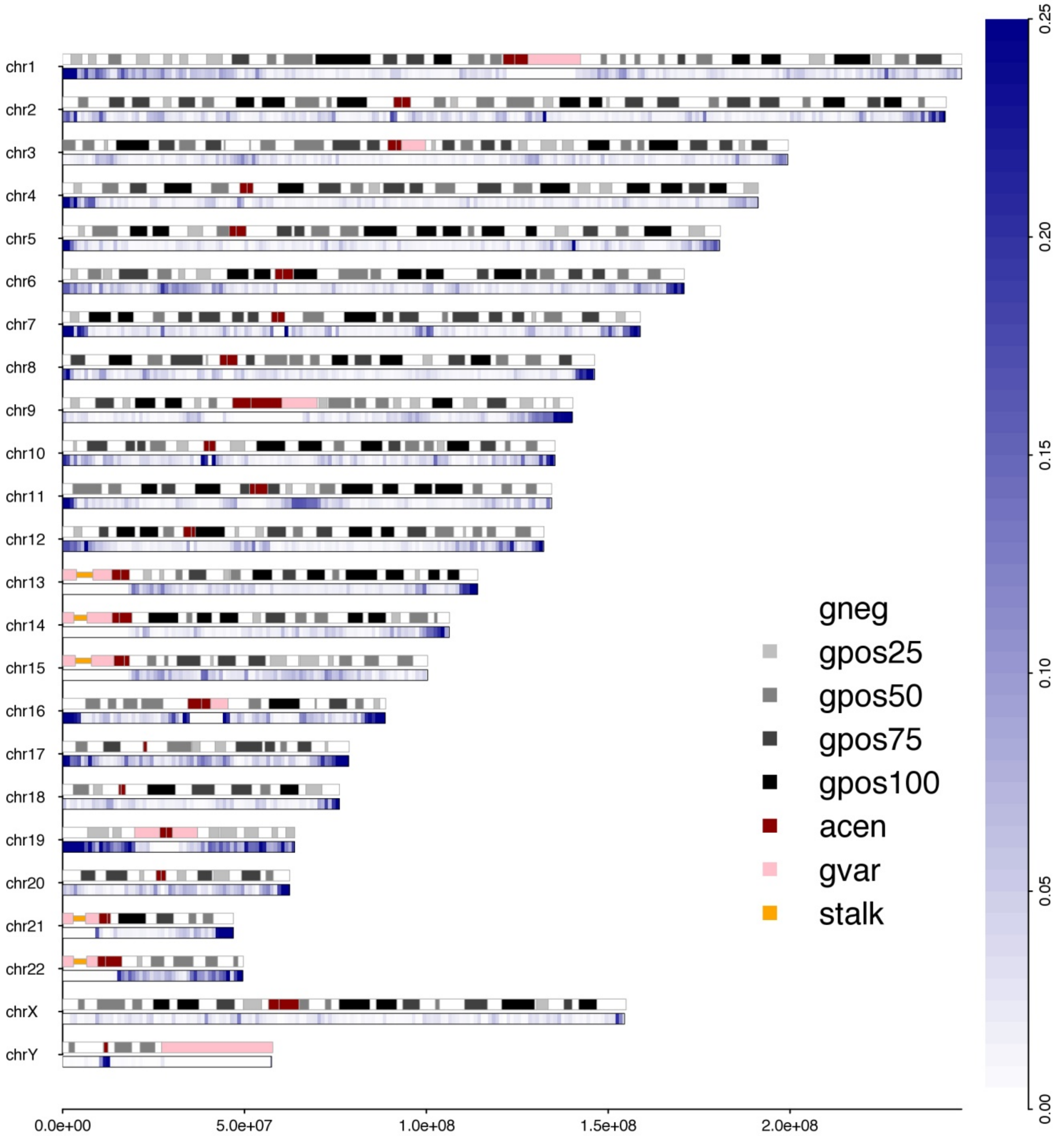

Supporting Figure S6: "Macroscopic" chromosomal STAMP patterns. STAMP density is represented in shades of blue for each chromosome (scale on right border). Plotted above each STAMP density is a chromosome ideogram with shades of grey representing the Giemsa staining intensity: negative, 25th, 50th, 75th, and 100th percentiles. Also indicated with colors are the centromere, variable regions and stalk as indicated in the legend.

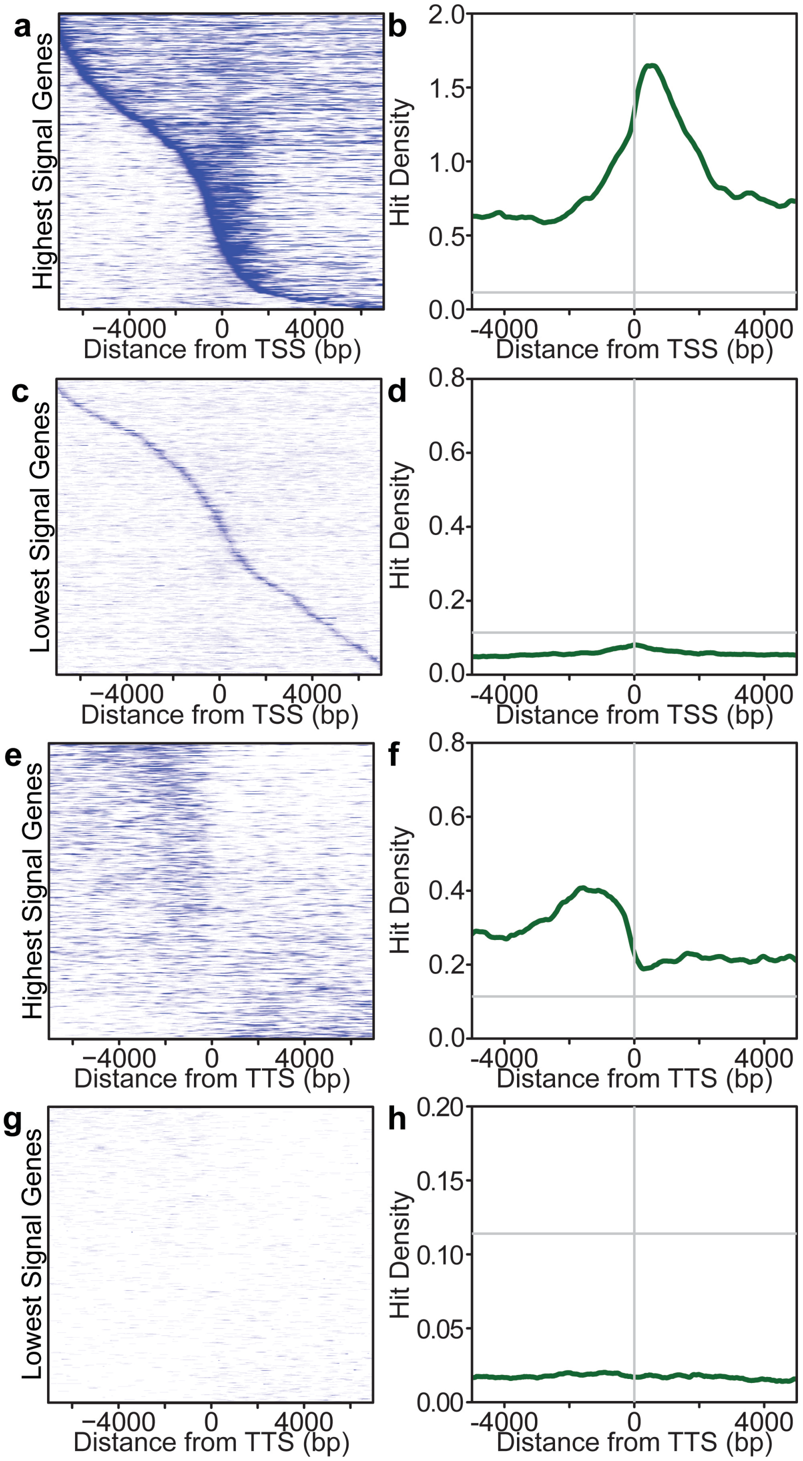

Supporting Figure S7: STAMP signal patterns for genes with the highest or lowest detectable methylation surrounding the TSS and TTS of all refGenes. STAMP signal for M091 cells was calculated for each TSS or TTS flanked by ~15 kb. Heatmaps representing the STAMP signal surrounding the TSS (**a, c**) or TTS (**e, g**) are shown for genes with either a (**a, e**) high STAMP signal (top 10% of total signal summed across the entire region) or a low signal (**c, g**). Rows are ordered by the location of mode and blue level is proportional to STAMP methylation. Each row represents an individual gene TSS and columns represent distance from the TSS or TTS, as indicated. Composite methylation profile (density across heatmap columns) for the genes in each heatmap is shown in the panels on the right (**b, d, f, h**).

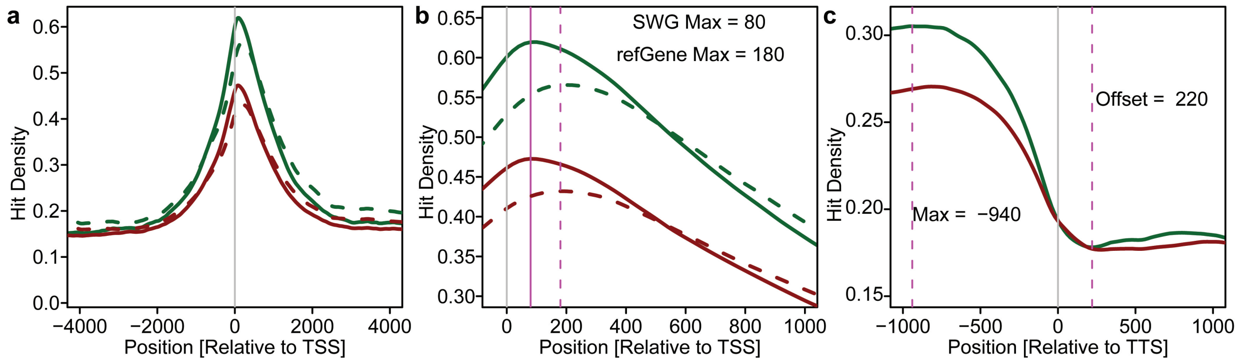

Supporting Figure S8: STAMP methylation offset at the TSS and the TTS. (**a**) The composite STAMP signal for M091 cells at the TSS for all refSeq genes (refGene) and for high-confidence TSS annotations in the Switchgear database is shown in dashed and solid lines, respectively. STAMP signal was calculated for each TSS or TTS flanked by ~15 kb. Data are shown for both untreated cells (green line) and decitabine-treated cells (red line). (**b**) The TSS methylation peak offset is shown for the composite profiles described in (a). (**c)** The TTS methylation peak offset is shown for the composite profiles of all refGenes for both untreated (green line) and decitabine-treated cells (red line).

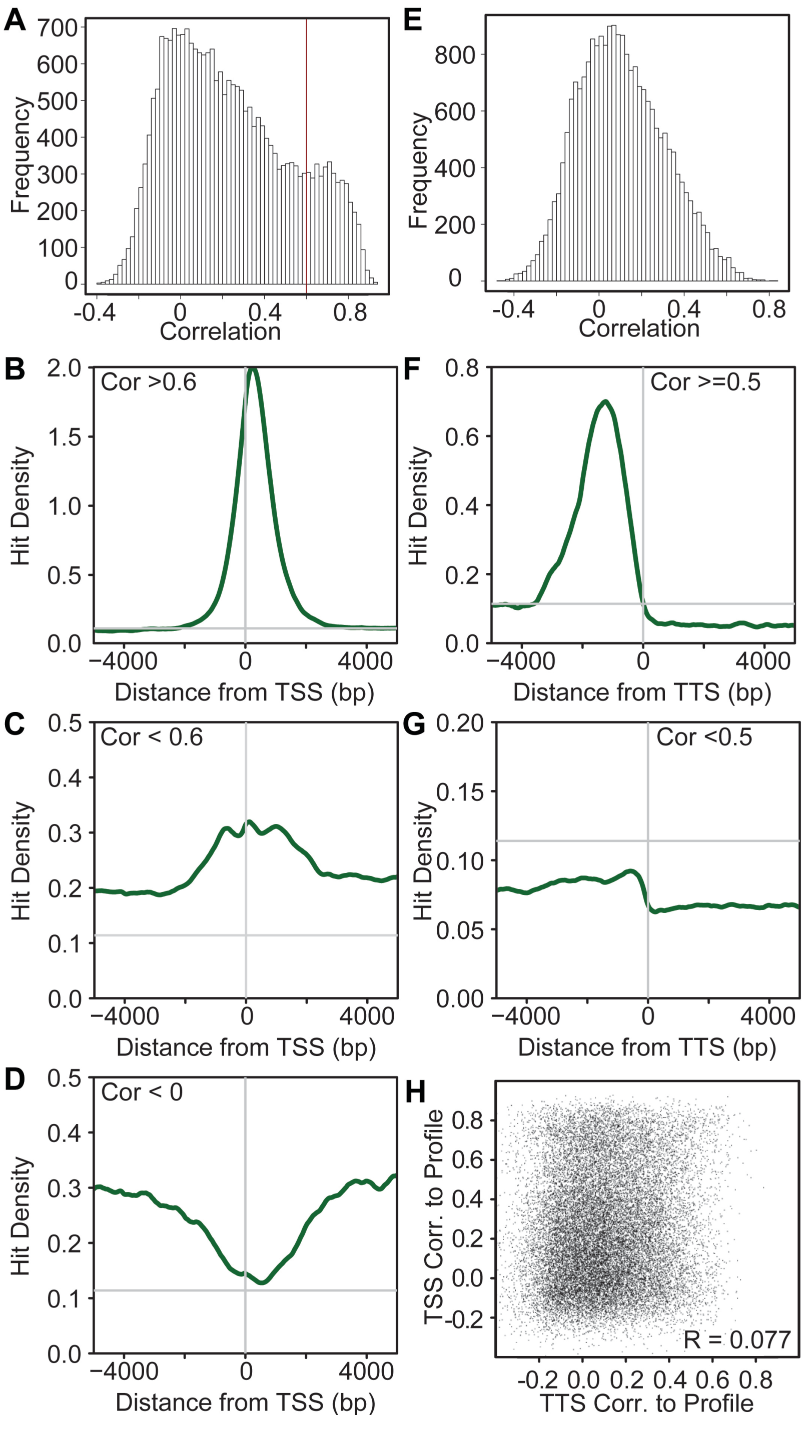

Supporting Figure S9: Patterns of DNA methylation at TSS and TTS. A STAMP signal was calculated for each TSS or TTS flanked by ~15 kb. (**A-D**) The similarity of each genes profile at the TSS compared to the composite profile (Fig 4a) using a Spearman correlation. (**A**) The histogram of these correlations has a bimodal distribution with a breakpoint ~0.6. (**B**) Genes with a correlation ≥ 0.6 have a strong methylation signal with a peak just downstream from the TSS. The tails extend below the noise floor. (**C**) Genes with a correlation < 0.6 have a more diffuse methylation of lower intensity. (**D**) Approximately 25% of genes have a negative correlation and reveals a STAMP signal pattern with tails of methylation above the noise floor extending into the gene body. (**E-G**) The similarity of each refGene profile at the TTS (Fig 4b) compared to the composite profile using a Spearman correlation. (**E**) The histogram of these correlations has a unimodal distribution. (**F**) Genes with a correlation ≥0.5 have a STAMP signal that peaks upstream of the TTS and reaches a minimum just after the TTS. (**G**) Genes with a correlation < 0.5 have a more diffuse methylation of low intensity. (**H**) The correlation between the TSS or the TTS and their respective composite densities was calculated for each gene and plotted. Each point represents a single gene. No linkage is apparent between the TSS and TTS profiles.

Decitabine-Regulated Transcripts

Gene Ontology Category	Adjusted p-val	Count	Regulation
response to wounding	1.49E-06	18	Up
death	3.46E-06	51	
cell motion	3.46E-06	31	
response to hypoxia	6.91E-04	10	
vasculature development	9.84E-04	17	
response to virus	9.84E-04	13	
negative regulation of developmental process	1.47E-03	24	
programmed cell death	1.47E-03	31	
immune response	1.84E-03	34	
regulation of body fluid levels	6.22E-03	12	
positive regulation of programmed cell death	6.33E-03	19	
ion homeostasis	8.02E-03	19	
rRNA processing	2.88E-03	7	Down
tRNA metabolic process	3.02E-03	8	
nitrogen compound metabolic process	7.51E-03	13	
ribosome biogenesis	7.51E-03	4	

Supporting Figure S10: Gene ontology of decitabine-regulated transcripts.

SUPPORTING REFERENCES

1. Weisenberger, D.J. et al. Analysis of repetitive element DNA methylation by MethyLight. *Nucleic Acids Res* **33**, 6823-6836 (2005).
2. Eckhardt, F. et al. DNA methylation profiling of human chromosomes 6, 20 and 22. *Nat Genet* **38**, 1378-1385 (2006).
